# Decreased fecal calprotectin levels in Spondyloarthritis patients colonized by *Blastocystis* spp.

**DOI:** 10.1038/s41598-022-18308-3

**Published:** 2022-09-23

**Authors:** Jacqueline Chaparro-Olaya, Liliana Morales, Moisés David León Falla, Paula C. Hernández, Wilson Bautista-Molano, Alejandro Ramos-Casallas, Juliette de Ávila, Juan Manuel Bello-Gualtero, Fabián Cortés Muñoz, Consuelo Romero-Sánchez

**Affiliations:** 1 Laboratorio de Parasitología Molecular, Vicerrectoría de Investigaciones, Universidad El Bosque, Bogotá, Colombia; 2 Cellular and Molecular Immunology Group (InmuBo), Universidad El Bosque, Bogotá, Colombia; 3 Cellular and Molecular Immunology Group (InmuBo), Universidad El Bosque. Clinical Immunology Group, School of Medicine, Hospital Militar Central, Bogotá, Colombia; 4 Rheumatology and Immunology Department & Clinical Immunology Group, School of Medicine, Hospital Militar Central, Bogotá, Colombia; 5 Vicerrectoría de Investigaciones, Universidad El Bosque, Bogotá, Colombia; 6 Cellular and Molecular Immunology Group InmuBo, Universidad El Bosque, Rheumatology and Immunology Department & Clinical Immunology Group, School of Medicine, Hospital Militar Central, Bogotá, Colombia; 7 Present Address: Laboratorio de Parasitología Molecular, Instituto de Biología Molecular, Universidad de El Bosque, Edificio O. Segundo piso, Avenida Cra. 9 No, 131 A – 02, Bogotá, Colombia

**Keywords:** Spondyloarthritis, Parasitic infection

## Abstract

Spondyloarthritis (SpA) is a group of chronic inflammatory systemic diseases mainly characterized by inflammation in the spine and/or peripheral joints. Although a link between SpA-pathogenesis, intestinal inflammation and gut dysbiosis has been proposed, studies have been focused on bacteria-host interactions and very little has been reported regarding intestinal parasites. Here, intestinal parasitic infection of 51 SpA-patients were evaluated and compared to healthy control individuals. No significant differences in the frequency of any parasite between SpA-patients and control individuals were found. Significantly higher levels of fecal calprotectin (FCP) were found in the SpA-patients compared to the control individuals. However, FCP levels were the same when comparing SpA-patients and control individuals, both colonized by *Blastocystis* spp. On the other hand, when comparing *Blastocystis* spp. colonized and *Blastocystis* spp. free SpA-patients, FCP levels were significantly higher in those *Blastocystis* spp. free. Without ignoring the small sample size as a study limitation, the results showed that in the SpA-patients colonized by *Blastocystis* spp., the FCP levels were significantly lower than those in the *Blastocystis* spp. free group and comparable to those in the control group. These findings seem to suggest a relationship between *Blastocystis* spp. and intestinal inflammation in SpA-patients, but studies intended to explore that interaction specifically should be designed.

## Introduction

Spondyloarthritis, (SpA) is a family of chronic inflammatory joint diseases. The main symptoms experienced by SpA-patients are inflammatory back pain and/or asymmetrical arthritis. In some rare cases, uveitis or enthesitis may be the primary manifestation. According to the 2009 Assessment in Spondyloarthritis International Society (ASAS) classification^1^, SpA-patients can be diagnosed with axial SpA (ankylosing spondylitis [AS] is the most studied of this group) or with peripheral SpA (which includes reactive arthritis [ReA], psoriatic arthritis [PsA], arthritis associated with inflammatory bowel disease [IBD], undifferentiated SpA [uSpA] and juvenile SpA [JSpA])^2^.

Gastrointestinal manifestations are frequent in SpA-patients and gut inflammation is one of the most important ones. The research on interaction between intestinal inflammation and arthritis was derived from demonstrating a high prevalence of ileitis or colitis in SpA-patients: 80% in JSpA, 65% in ReA, 65% in uSpA, 57% in AS and 16% in PsA^3,4^. Accordingly, a high risk of progression to AS has been observed in patients with chronic intestinal inflammation and diarrhea^5,6^ and intestinal inflammation is verifiable through endoscopic and histological examination in 40–60% of AS-patients^7^. Likewise, a higher prevalence of inflammatory bowel disease (IBD) in AS-patients has also been described, compared to healthy control individuals^5,8^. Although the exact pathogenic pathway by which concomitant inflammation occurs in the gut and joints is unknown, it has been suggested that intestinal inflammation alters the immune system and intestinal permeability, which allows antigens and activated macrophages to enter into circulation and produce joint inflammation^9,10^.

On the other hand, although there is evidence of a relationship between intestinal inflammation and SpA pathogenesis, the role of the microbiome in that interaction is still unclear. In fact, there are very few reports in this regard and most of them explore the relationship between certain types of bacterial gut infections and AS. Although some studies have not found disturbances of the main bacterial groups in the fecal microflora of AS-patients (*Bacteroides, Eubacterium-Clostridium* and *Bifidobacterium*)^11^, Costello et al. demonstrated a relationship between disease status and bacterial community composition when comparing AS-patients to control individuals. Therefore, the authors presented the first characterization and identification of intestinal dysbiosis in the AS microbiome^12^. At the same time, the association between the disease and *Klebsiella pneumoniae* (which was proposed initially as the main microbial trigger and/or perpetuator of AS) has been investigated exhaustively, with contradictory results^13^. The picture is more complex when considering that gut microbiota studies in SpA-patients have focused on bacteria-host interactions and very little has been reported regarding intestinal parasites. There are only a few known case reports that point to *Blastocystis* spp.^14,15^, *Cryptosporidium*^16,17^, *Dracunculus*^18^, *Endolimax nana*^19^, *Entamoeba hartmanni*^20^, *Entamoeba histolytica*^19,21^, *Giardia intestinalis*^22–24^, *Strongyloides stercoralis*^19,25,26^ and *Taenia saginata*^19,25^ as causal agents of ReA. Some reports propose *Toxoplasma gondii* as a triggering agent of ReA^19^ but more recent studies suggest these findings may be coincidental^27^. As far as we know, there are only a few studies on intestinal parasitosis and SpA, and all of them focus on ReA: the aforementioned study on *T. gondii*^27^, another one on *G. intestinalis*^28^ and another on *Giardia* in a retrospective and large health insurance cohort^29^.

The aim of this work was to analyze intestinal parasitosis in a group of SpA-patients and to evaluate its relationship with intestinal inflammation. Because fecal calprotectin (FCP) is released during neutrophil activation^30^ and reflects the increased migration of these cells into the intestinal lumen and through the inflamed mucosa, FCP levels are high in patients with active inflammatory bowel diseases^31^. Additionally, several studies have shown this protein is elevated in SpA-patients and IBD-patients, and that it correlates well with histological inflammation detected by colonoscopy^31–38^. Therefore, FCP was used as a marker of inflammation.

## Results

### Population

Fifty-one SpA-patients and 50 control individuals were included in the study. Among the SpA-patients, 86.27% (n = 44) were AS-patients, 7.84% (n = 4) uSpA-patients and 5.89% (n = 3) PsA-patients. Regarding clinical expression, 82.35% (n = 42) had axial involvement, 11.76% (n = 6) peripheral and 5.89% (n = 3) mixed. In all, 96.08% (n = 49) were receiving treatment, and ASDAS-CRP and ASDAS-ESR disease activity indices both showed active disease in 90.2% of the cases. The demographic and clinical characteristics of the participants are summarized in Table 1.

**Table 1:** Demographic and clinical characteristics of SpA-patients and control subjects.

Variable	SpA-patients (n = 51)	Control subjects (n = 50)	*p* value
**Sex (n–%)**			
Female	21–41.18	23–46.00	0.6250*
Male	30–58.82	27–54.00	
Age (years) Median (IR)	43 (36–53)	38 (29–52)	0.0701**
FCP (ng/ml) Median (IR)	53.9 (43.89–98.88)	44.64 (41.9–56.8)	0.0082**
**Treatment (n–%)**			
None	2–3.92		
NSAIDs	9–17.65		
cDMARDs (Sulfasalazine/Methotrexate)	5–9.80		
bDMARDs. Anti-IL17 (n = 4)	35–68.63		
bDMARDs. Anti-TNFα (n = 31)			
**SpA markers**			
CRP (mg/dL) Median (IR)	0.37 (0.15–2.23)		
ESR (mm/h)			
General Median (IR)	10 (5–25)		
Female Median (IR)	25 (14–30)		
Male Median (IR)	5 (3–11)		
BASDAI Mean (SD)	4.58 (2.46)		
**BASDAI (n–%)**			
Without active difficulty	24–47.06		
With active difficulty	27–52.94		
BASFI Mean (SD)	4.22 (2.81)		
BASFI (n–%)			
Without active difficulty	25–49.02		
With active difficulty	26–50.98		
ASDAS CRP Mean (SD)	2.41 (0.80)		
**ASDAS CRP scores (n–%)**			
Inactive disease	5–9.8		
Moderate activity	11–21.57		
High activity	30–49.02		
Very high disease activity	5–9.8		
ASDAS-ESR Mean (SD)	2.63 (0.98)		
**ASDAS-ESR scores (n–%)**			
Inactive disease	5–9.8		
Moderate activity	8–15.69		
High activity	26–50.98		
Very high disease activity	12–23.5		
**Gastrointestinal symptoms (n–%)**			
Presence of symptoms	38–74.51		
Diarrhea lasting more than 4 weeks	25–49.02		
Blood in stool	8–15.69		
Mucus in stool	12–23.53		
Abdominal pain	34–66.67		
Weight loss	9–17.65		
Abdominal distention	33–64.71		
Bowel movements per day. Median (IR)	1 (1–3)		

SD, standard deviation. IR, interquartile range. FCP, fecal calprotectin. SpA, Spondyloarthritis. CRP, C-reactive protein. ESR, erythrocyte sedimentation rate. NSAIDs, non-steroidal anti-inflammatory agents. cDMARDs, conventional disease modifying anti-rheumatic drugs. bDMARDs, biological disease modifying anti-rheumatic drugs. (*) Z test of proportion differences. (**) Wilcoxon rank-sum test.

### FCP and clinical characteristics

Elevated FCP levels (≥ 120 ng/ml) were found in 21.57% (n = 11) of the SpA-patients and 14.0% (n = 7) of the control individuals. The median FCP concentration was significantly higher in the SpA-patients compared to the control group (p = 0.0082) (Table 1). Analyzing the subgroup of SpA-patients who had elevated FCP versus the subgroup of SpA-patients with normal FCP (< 120 ng/ml), gastrointestinal symptoms were present in 80.0% of the former group and 81.82% of the latter. On the other hand, according to the limit values established for CRP (C-reactive protein) and ESR (Erythrocyte Sedimentation Rate) (< 3 mg/L and < 20 mm/h, respectively), 11.76% (n = 6) of the SpA-patients had elevated values for CRP, 31.37% (3 men and 13 women) for ESR and 7.84% (n = 4) for both. Among SpA-patients, 52.94% (n = 27) had normal values for FCP, ESR and CRP simultaneously, and none had all three markers elevated at the same time. Finally, Spearman correlation coefficients showed that FCP was positively correlated with ESR, BASDAI and ASDAS-ESR, and had no correlation with CRP, BASFI and ASDAS-CRP (Table 2).

**Table 2:** Correlations between FCP and CRP, ESR, BASDAI, BASFI and ASDAS in SpA-patients.

	FCP (Spearman’s correlation)		
	Rho	Strength	*p* value
CRP	0.1799	Poor	0.2065
BASFI	0.2089	Poor	0.1412
ASDAS- CRP	0.2615	Poor	0.0638
BASDAI	0.3060	Fair	0.0290
ESR	0.3171	Fair	0.0234
ASDAS- ESR	0.3495	Fair	0.0119

FCP: fecal calprotectin. CRP: C-reactive protein. ESR: erythrocyte sedimentation rate. Spearman’s correlation coefficient was calculated and the strength of correlation (Strength) was classified as poor (zero < rho < 0.3) and fair (0.3 ≤ rho < 0.6)^39^.

### Intestinal parasite infection

There were no significant differences in the frequency of any parasite between SpA-patients and control individuals and none of the samples were positive for helminths (Table 3. Supplementary Fig. S1). No parasitic species was exclusive to any of the groups, except *Iodamoeba bütschlii*, which only occurred in one control individual (Supplementary Table S1). The qPCR assay detected five (5) other *Blastocystis* spp. positive samples in addition to those detected by microscopy. In the case of *G. intestinalis*, qPCR revealed only one additional infection. *Entamoeba histolytica* PCR revealed six (6) infections in addition to the three (3) detected for the *Entamoeba complex* by microscopy (Table 3). For 27.45% of the SpA-patients and 21.57% of the control individuals, *E. nana* was the only parasite identified. Dual colonization with *E. nana* and *Blastocystis* spp. occurred in 51% of the SpA-patients and 56% of the control individuals. The next most frequent combination was *E. nana, Blastocystis* spp. and *Entamoeba coli,* which occurred in 7.84% of the SpA-patients and 10% of the control individuals (Supplementary Table S1).

**Table 3:** Intestinal parasites found in SpA-patients and control subjects.

	Diagnostic method	Total	SpA-patients (n = 51)		Control subjects (n = 50)		*p* value
		n	n	%	n	%	
**Parasite**							
*Chilomastix mesnili*	Microscopy	3	2	3.92	1	1.96	0.5579*
*Endolimax nana*	Microscopy	101	51	100.0	50	100.0	–
*Entamoeba coli*	Microscopy	12	5	9.80	7	13.73	0.5388*
*Iodamoeba bütschlii*	Microscopy	1	0	0	1	1.96	0.3149*
*Entamoeba* complex ^**1**^	Microscopy	3	1	1.96	2	4.00	0.5461*
*Entamoeba histolytica* ^**1**^	PCR	6	4	7.84	2	3.92	0.4000*
*Blastocystis* spp.	Microscopy and qPCR	73	34	66.67	39	76.47	0.2724*
*Giardia intestinalis*	Microscopy and qPCR	3	1	1.96	2	3.92	0.5579*

(1): Because *E. histolytica* cannot be morphologically differentiated from *E. dispar* and *E. moshkovskii* by microscopic examination, a PCR assay was done to identify *E. histolytica*. Microscopic examination revealed the presence of *Entamoeba* complex in three samples. The *E. histolytica* PCR was negative for these three samples, but revealed six additional infections. (*) Z test of proportion differences.

In summary, all the participants were infected with at least one of eight intestinal protists (Table 3). Because *E. nana* was present in 100% of the population, this parasite was not taken into account in the analyses of association with clinical characteristics. Parasites that were present in a very low proportion, or as co-infection with any other intestinal parasite different from *E. nana*, were also not included. Therefore, *I. bütschlii, G. intestinalis, C. mesnili, Entamoeba coli, E. histolytica* and the *Entamoeba* complex were not included in the analysis (Table 3 and Supplementary Table S1). The only parasite that met the requirements for analysis was *Blastocystis* spp.

### *Blastocystis* spp

Although it was established that there was a significant difference in FCP levels between the SpA-patients and the control individuals (*p* = 0.0082), analysis showed no significant difference when *Blastocystis* spp. colonization was taken into account. To perform this analysis, individuals without *Blastocystis* spp. (individuals not infected with any parasite, except *E. nana*) and individuals with *Blastocystis* spp. (those who, in addition to *E. nana*, had *Blastocystis* spp. as the only co-infecting parasite) were compared. Although a significant difference in FCP levels was still observed between SpA-patients and the control group in individuals without *Blastocystis* spp., there was no difference when individuals with *Blastocystis* spp. were compared (Table 4).

**Table 4:** Fecal calprotectin levels in *Blastocystis* spp. free and *Blastocystis* spp. colonized individuals: Comparison between SpA-patients and healthy control subjects.

	SpA-patients (n = 51)		Control subjects(n = 50)		*p* value
**Fecal Calprotectin (ng/ml). Median (IR)**					
Without *Blastocystis* spp.	n = 14	78.3 (50.75–208.36)	n = 9	44.0 (42.0–46.7)	0.0136**
With *Blastocystis* spp.	n = 26	57.06 (43.5–89.03)	n = 28	43.75 (41.0–55.0)	0.0664**

IR, interquartile range. (**) Group comparisons were performed using a Wilcoxon rank-sum test. Bonferroni correction was applied to account for multiple comparisons.

Additionally, when comparing median FCP concentrations among the SpA-patients, significantly lower values were observed in those colonized by *Blastocystis* spp. In the control group, no such difference was observed between *Blastocystis* spp. colonized and *Blastocystis* spp. free individuals (Table 5).

**Table 5:** Fecal calprotectin levels in SpA-patients and control subjects: Comparison between *Blastocystis* spp. free and *Blastocystis* spp. colonized individuals.

	Without *Blastocystis* spp.		With *Blastocystis* spp.		*p* value
**Fecal Calprotectin (ng/ml). Median (IR)**					
SpA-patients (n = 40)	n = 14	78.3 (50.75–208.36)	n = 26	57.06 (43.5–89.03)	0.0036**
Control subjects (n = 37)	n = 9	44.0 (42.0–46.7)	n = 28	43.75 (41.0–55.0)	0.3684**

IR, interquartile range. (**) Group comparisons were performed using a Wilcoxon rank-sum test. Bonferroni correction was applied to account for multiple comparisons.

Among the SpA-patients colonized by *Blastocystis* spp. 15.38% had elevated CRP and 34.61% had elevated ESR. In the SpA-patients without *Blastocystis* spp., these frequencies were similar (14.28% and 35.71%, respectively). Additionally, in the SpA-patients with *Blastocystis* spp. versus those without *Blastocystis* spp., there were no significant differences in median values for CRP [0.64 mg/L (0.22–2.46) versus 0.43 mg/L (0.17–2.45) (*p* = 0.0842)] or ESR [10.0 mm/h (4.25–25) versus 14.5 mm/h (9.25–23.75) (*p* = 0.8723)]. Regarding gastrointestinal symptoms, 85.71% of the SpA-patients colonized by *Blastocystis* spp. and 76.92% of the SpA-patients without *Blastocystis* spp. reported at least one gastrointestinal symptom. None of gastrointestinal complaints could be linked to *Blastocystis* spp. (Supplementary Table S2).

## Discussion

In this work, clinical characteristics of 51 SpA-patients and the frequency of intestinal parasitosis in them, were evaluated. As noted in the introduction section, research on the associations between SpA and intestinal microbiome has focused mainly on an analysis of prokaryotes, while information on parasites has been limited to a few case reports of ReA^14–26^.

In this study, 100% of the participants (51 SpA-patients and 50 control individuals) showed infection with some intestinal protist and none of them with helminths (Table 3). There were no significant differences in the frequency of any parasite between the SpA-patients and the control individuals, and the most prevalent parasite in both groups was *E. nana*, followed by *Blastocystis* spp.

Although there are some studies that have proposed *E. nana* as a cause of diarrhea, these findings have been debated for reasons including lack of evidence regarding co-infection with other intestinal microorganisms causing this symptomatology, and even misdiagnosis of *E. nana*^40^. Similarly, a study that proposed *E. nana* as a cause of parasitic rheumatism^41^ also was refuted because no tests were done to diagnose the reported ReA^40^. We concur with the opinion that *E. nana* does not cause intestinal inflammation per se. Coupled with the fact that it was present in 100% of the participants, it meant this parasite was not taken into account in the analyses of association with demographic or clinical characteristics. It is relevant to note that there is very little research focused specifically on the study of *E. nana* and, in general, the reported prevalences correspond to findings in studies focused on other parasites or to general searches for intestinal parasitosis. In fact, a search for “*Endolimax nana* [title]” in PubMed (on the 15th of July 2022) identified only 15 articles and most of them are relatively old. In Colombia, high frequencies of *E. nana* colonization were reported when simultaneous diagnosis is made by direct examination and by concentration (for example, 60% in Bermúdez et al.^42^ and 77.35% in Hernández et al.^43^). However, these were local studies on minority groups or schoolchildren from vulnerable communities. Furthermore, when comparing epidemiological data from different studies, not only the diagnostic method and the target population should be considered, but also the period when the surveillance was carried out. For example, a retrospective study in Chile involving 68.142 preschool and school children showed that *E. nana* decreased from 26.26% in 1980 to 10.14% in 2007^44^. However, a later Chilean study in 103 students from urban and rural schools found an *E. nana* prevalence of 40.2%^45^. A similar result (41.2%) was obtained among 119 healthy food handlers from Zulia, Venezuela^46^. In Brazil, a study conducted in 3,245 individuals (aged 1 to 93 years) attending the FIOCRUZ Institute identified *E. nana* as the most common enteric parasite (6.66%)^47^. The same in 595 children and adults living in Brazilian urban slums in whom *E. nana* was detected with a prevalence of 16%^48^. In the United States, 2,896 patients from throughout the country were tested for intestinal parasites and *E. nana* was detected in 46 participants (1.6%)^49^. A similar prevalence was found (1.5%) in 2,604 patients from the states of Colorado, Montana, Utah, and New Mexico^50^. In Algeria, *E.* nana was found in 233 out 2,054 children and adult patients (11.34%)^51^, while the frequency of the parasite was found to be much higher (63.4%) in 93 school attendees in Zambia^52^. In Iran, in 1,025 stool samples collected from 50 villages, *E. nana* prevalence varied from 18.18% in the youngest (1–10 years) to 34.04% in the group over 50 years of age^53^. In Europe, a study in areas with poor sanitation in Slovakia analyzed 2,760 individuals aged 1 month to 88 years and revealed 16 (0.58%) positive samples for *E. nana*^54^. Similarly, in a retrospective study in 572 Swiss children attending a hospital, *E. nana* was the second most frequent parasite (0.5%)^55^. Considering the available background on the epidemiology of *E. nana*, it is not possible to estimate the frequency of this parasite in the study population (SpA-patients and healthy adults from urban areas with socioeconomic conditions comparable to those of developed countries) and to try to explain the high prevalence found.

Regarding *Blastocystis* spp.*,* it is also difficult to compare the frequency found in this study to those in earlier reports, since studies have shown that *Blastocystis* spp*.* prevalence is highly dependent on the population studied and techniques employed for diagnosis. For example, a literature search on reports of *Blastocystis* spp. in Brazil between 2000 and 2020 revealed frequencies between 0.5% and 86.6% in the 52 studies ultimately included in the analysis^56^. Similarly, a study in China from 1990 to 2019 found prevalences of *Blastocystis* spp. in a range of 0.04% to 43.26%^57^. In the present study, the prevalence of *Blastocystis* spp. was 66.67% in the SpA-patients and 76.47% in the control individuals, which did not represent a significant difference. The high prevalence of *Blastocystis* spp. in healthy adult groups (even in industrialized countries) has already been reported^58,59^, as well as its higher prevalence in control groups compared to patients with intestinal inflammation^60–62^.

As for FCP, its concentration was significantly higher in the SpA-patients compared to the control individuals. Similar findings have been previously reported^34–38^. Simultaneously, in the SpA-patients, a positive and significant correlation was observed between FCP/ESR, FCP/BASDAI and FCP/ASDAS-ESR, but not between FCP/CRP, FCP/BASFI or FCP/ASDAS-CRP (Table 2). No relationship was found between the level of FCP and gastrointestinal symptoms.

On the other hand, when analyzing FCP in individuals without *Blastocystis* spp., the result was the same as that found in the general population: FCP concentration was significantly higher in the SpA-patients than in the control individuals. However, when SpA-patients and control individuals, both colonized by *Blastocystis* spp. were compared, there was no difference in FCP levels (Table 4). Likewise, when comparing *Blastocystis* spp. colonized and *Blastocystis* spp. free SpA-patients, FCP levels were significantly lower in those colonized. This difference was not observed in the control group (Table 5). This behavior of the control group differed with the findings of Nieves-Ramirez et al. who enrolled healthy individuals free of gastrointestinal disorders and measured FCP in subgroups of subjects who were positive and negative for *Blastocystis* spp. They showed that FCP levels in all the individuals were well below levels compatible with clinical inflammation, but were significantly lower in those colonized by *Blastocystis* spp.^63^. More recently, Ibrahim et al. analyzed stool samples from children and showed that colonization with *Blastocystis* spp. had no significant effect on FCP levels^64^. In the face of contradictory evidence, we believe these findings may not be comparable due to the diverse aims and approaches of the studies and the differences in experimental designs and sociodemographic characteristics of the study populations. In addition, the existence of indirect factors that have not been contemplated and could influence these findings cannot be ruled out. Therefore, studies aimed at specifically analyzing the effect of *Blastocystis* spp. colonization on intestinal inflammation in SpA-patients are needed.

In a recent study in which rats were inoculated with *Blastocystis* cysts (isolated from feces of an asymptomatic human donor) and colitis was induced to monitor the intensity of inflammation compared to a control group, it was found that long-term colonization with *Blastocystis* spp. was associated with a more severe initial response but a more rapid recovery from colitis^65^. Although it will have to be confirmed that these findings can be translated to humans, they suggest that long-term colonization with *Blastocystis* spp. may be a protective factor by promoting a more rapid resolution of intestinal inflammation.

With regard to ESR and CRP, no differences in the values of these parameters were observed between SpA-patients with and without *Blastocystis* spp. Similarly, Coskun et al. compared *Blastocystis* spp. colonized and *Blastocystis* spp. free ulcerative colitis patients and found no significant differences in ESR and CRP values^66^. In contrast, Javaherizadeh et al. reported significantly higher ESR values in individuals colonized by *Blastocystis* spp. (11.16 ± 8.67 versus 10.32 ± 5.01. *p* < 0.01). However, the study included subjects who went to hospitals for routine checkups and does not state or analyze the existence of any underlying disease or clinical condition^67^. The authors also found elevated CRP values in colonized individuals more frequently (1.46%) than in non colonized individuals (0.5%), but did not report median concentration values for each group.

Finally, it is necessary to remark the potential influence of drug therapy on FCP levels. In this study, 68.63% of the SpA-patients received treatment with bDMARDs (Anti IL-17 or Anti TNF-α), 17.65% with NSAIDs and 9.80% with cDMARDs (sulfasalazine or sulfasalazine and methotrexate). As for NSAIDs, their gastrointestinal toxicity has been widely reported. It is known they can increase intestinal permeability within hours of administration and trigger small bowel inflammation days later. In the long term, they can lead to a condition known as NSAID-induced enteropathy, which causes elevation of inflammatory markers such as FCP^68^. Even so, it is unlikely these drugs contribute significantly to the etiology of inflammation in SpA, as NSAID enteropathy is not usually localized in the ileum or colon^4,69^. Along the same line, a study in Spain with 33 SpA-patients found that treatment with NSAIDs did not modify FCP levels^37^. Likewise, a study in Brazil with 85 SpA-patients^31^ and another in Croatia with 61 children who were diagnosed with JSpA^70^ found that median FCP concentrations were the same between patients who received NSAIDs or DMARs and those who did not. The foregoing leads to the assumption that the observed differences in FCP levels or parasite colonization within the SpA-patients group are unlikely to be the result of treatment. Other authors such as Klingberg et al. showed that FCP levels in AS-patients increased as the frequency of NSAID intake increased, and discontinuation of the drug for three weeks led to a drop in FCP values. However, the authors pointed out that this effect was not due solely to the use of this type of medication, since they found elevated FCP levels in 63% of the patients who did not use NSAIDs or did so only a few times a year^34,36^. Similarly, these authors found significantly lower FCP values in patients receiving methotrexate and infliximab treatment, but not so in those receiving etanercept or sulfasalazine^34,36^. The findings for adalimumab were conflicting^34,36^. Although the number of individuals is not sufficient to make a conclusive comparison, in this study we found no difference in median FCP values between the SpA-patients receiving anti-TNF- (n = 31), NSAIDs (n = 9) or sulfasalazine (n = 3) treatment: 49.70 ng/ml, 49.97 ng/ml and 45.65 ng/ml, respectively.

The study limitations included the small sample size and the fact that we are unable to establish whether *Blastocystis* spp. can actually influence FCP concentration in SpA-patients, or whether an unknown intestinal context promotes the presence of *Blastocystis* spp. and reduced inflammation. A study specifically focused on *Blastocystis* spp. is needed, which also takes into account the intestinal microbiome and the possible influence of therapeutic treatment.

To our knowledge, this is the first study that simultaneously analyzes the parasitic intestinal population and the clinical characteristics of SpA-patients. Without ignoring the aforementioned study limitations, the results showed that in the SpA-patients colonized by *Blastocystis* spp., the FCP levels were significantly lower than those in the *Blastocystis* spp. free group and comparable to those in the control group. Although the data seem to suggest a relationship between *Blastocystis* spp. and intestinal inflammation in SpA-patients, studies intended to explore that interaction specifically should be designed.

## Methods

### Study population

A group of individuals with diagnosis of SpA (SpA-patients) and a group of healthy individuals (control) were included in the sample. Participants were enrolled between April 2019 and February 2020. The SpA-patients were subjects older than 18 and younger than 65 years of age. They were selected by a group of experienced rheumatologists, according to the criteria of the ASAS group^1^, who questioned them about the presence of gastrointestinal symptoms (diarrhea, mucus in the stool, hematochezia, increased daily bowel movements, abdominal pain or abdominal distension). SpA-patients were enrolled from two health institutions in Bogota, Colombia (Hospital Militar Central and Clinicos IPS SAS). The control group consisted of healthy individuals residing in Bogotá, with similar age, profession and socioeconomic conditions, in whom the presence of joint and gastrointestinal symptoms was ruled out. Pregnant women or patients with a history of infection in the last month, autoimmune disease, neoplasia, immunodeficiency, chronic pancreatitis, liver disease or diabetes were not included. Participants who had taken any antibiotic or antiparasitic in the last three (3) months, or who were receiving any dose of systemic steroid at the time of inclusion in the study were also not included. The rheumatologic involvement of each patient was assessed using the following clinical indices: BASFI, BASDAI, ASDAS-CRP and ASDAS-ESR^71,72^. A stool sample was collected from each participant to diagnose intestinal parasitosis and to measure FCP. For participants who had a positive diagnosis for intestinal parasitosis, treatment was prescribed by a physician according to the infecting species. Additionally, a blood sample was collected from each patient to measure CRP and ESR.

### Ethics declarations

This study was conducted in accordance with the principles outlined in the Declaration of Helsinki and guidelines established by the Ministerio de Salud y Protección Social de Colombia for research involving human subjects. SpA-patients and control individuals made a voluntary decision about participating in the study and signed an informed consent for collection/use of data and samples. Ethics Committee of Hospital Militar Central in Bogotá, Colombia (records number 09 of May 5, 2017, and 09 of June 1, 2018. Code: 2017-023) approved this research.

### FCP, CRP and ESR measures

Quantitative measurement of FCP level was performed by ELISA based methods using KAPEPKT849 kit (DIAsource ImmunoAssays S.A. Louvain-la-Neuve, Belgium). Chemiluminescence immunoassay Immulite 1000 (Siemens Healthineers. Erlangen, Germany) and Westergren sedimentation rate testing were used on blood samples to measure CRP and ESR, respectively.

### Microscopic detection of intestinal parasites

Stool samples were analyzed by microscopic examination using three setups: wet mount (0.9% saline solution and 4% lugol), concentration with the Mini-Parasep SF system (DiaSys. Wokingham, UK) and Kato-Katz (Sterlitech. Washington, USA). Two bacteriologists with certified clinical experience read each mount.

### Molecular detection of ***E. histolytica***, ***G. intestinalis*** and ***Blastocystis*** spp

In addition to microscopy-based parasite identification, the presence of *E. histolytica* was assessed by PCR, while the presence of *G. intestinalis* and *Blastocystis* spp. was confirmed by quantitative PCR (qPCR).

First, DNA from ≈200 μl of concentrated stool samples was extracted using the QIAamp DNA Stool Mini Kit (Qiagen. Germantown, USA). Then, *Enterobacteriaceae* 16S rRNA gene-specific primers and a TaqMan probe (synthetized by Macrogen. Seoul, Korea) (Supplementary Table S3) were used to confirm successful DNA extraction by qPCR^73^. *Enterococcus faecalis* strain ATCC 29212 was used as positive control. The qPCR reactions were performed with TaqMan® Fast Advanced Master Mix (BioRad. Hercules, USA) and run on a CFX96 Touch real-time PCR system (BioRad. Hercules. USA).

Because *E. histolytica* cannot be morphologically differentiated from *E. dispar* and *E. moshkovskii* through microscopic examination, a semi-nested PCR protocol to amplify the 18S rRNA gene was done to identify *E. histolytica*^74,75^. Both PCR reactions were carried out in a mix containing 0,2 mM dNTPs, 2 mM MgCl2, 0.5 μM of each primer (synthesized by Macrogen. Seoul, Korea) (Supplementary Table S3) and DNA GoTaq® Flexi DNA Polymerase (Promega. Madison, USA). Reactions were performed on a T100 PCR thermal cycler (BioRad. Hercules, USA) as follows: 3 min at 94 °C and 35 cycles of 1 min at 94 °C, 1 min at 55 °C, and 1 min at 72 °C, with a final step of 7 min at 72 °C. As template, 3 μl of each DNA extraction were used in the first PCR and then 2 μl of the PCR products from the first step were used as templates for the second step. A plasmid containing the product of the first amplification was produced and used as positive control. The identity of the construct was confirmed by sequencing (Macrogen-Seoul, Korea).

*Giardia intestinalis* and *Blastocystis* spp. were identified by qPCR using the primers and TaqMan probes reported by Mejia et al.^75^ and Stensvold et al.^76^, respectively (synthesized by Macrogen. Seoul, Korea) (Supplementary Table S3). Reactions were performed individually for each parasite with 2 μl of DNA extraction as template and TaqMan® Fast Advanced Master Mix (BioRad. Hercules, USA). Programs were run on a CFX96 Touch Real-Time PCR System (BioRad. Hercules, USA) as in Mejia et al.^75^ and Stensvold et al.^76^. DNA of *G. intestinalis* (strain WB) and *Blastocystis* spp. (strain BT1) were used as templates in the qPCR reactions and the obtained products were cloned (vector pJET1.2blunt.Thermo Scientific™. Waltham, USA), sequenced (Macrogen. Seoul, Korea) and used as templates in calibration curves (dilutions between 10,000 fg/μL to 0.1 fg/μL).

### Statistical Analysis

Descriptive statistics, including mean, median, standard deviation (SD), and interquartile ranges, were used to characterize the study population according to the assumption of normality, as evaluated using the Shapiro–Wilk test. Group comparisons were performed using a mean-difference Z test to continuous variables conforming to a normal distribution; otherwise, a Wilcoxon rank-sum test was applied to other continuous variables. Dichotomic qualitative variables were compared between two groups using a Z test of proportion differences. Correlations between variables were evaluated by Spearman's correlation due to a non-normal distribution in those variables. When it was appropriate, Bonferroni correction was applied to account for multiple comparisons. A *p* value < 0.05 was considered statistically significant. Statistical analyses were performed using Stata V.12.1.

## Supplementary Information

Supplementary Information.

## Data Availability

The data underlying this article cannot be shared publicly due to the privacy of individuals that participated in the study. The data will be shared on reasonable request, and may be requested via email to romeromaria@unbosque.edu.co.

## References

[1] Sieper J (2009). The Assessment of Spondyloarthritis International Society (ASAS) handbook: A guide to assess spondyloarthritis. Ann. Rheum. Dis..

[2] Zeidler H, Amor B (2011). The Assessment in Spondyloarthritis International Society (ASAS) classification criteria for peripheral spondyloarthritis and for spondyloarthritis in general: the spondyloarthritis concept in progress. Ann. Rheum. Dis..

[3] Mielants H, Veys EM, Cuvelier C, De Vos M (1996). Course of gut inflammation in spondylarthropathies and therapeutic consequences. Baillieres. Clin. Rheumatol..

[4] Smale S, Natt RS, Orchard TR, Russell AS, Bjarnason I (2001). Inflammatory bowel disease and spondylarthropathy. Arthritis. Rheum..

[5] De Vos M, Mielants H, Cuvelier C, Elewaut A, Veys E (1996). Long-term evolution of gut inflammation in patients with spondyloarthropathy. Gastroenterology.

[6] Karreman MC, Luime JJ, Hazes JMW, Weel AEAM (2017). The prevalence and incidence of axial and peripheral spondyloarthritis in inflammatory bowel disease: a systematic review and meta-analysis. J. Crohns Colitis..

[7] Kontny E, Dmowska-Chalaba J (2019). Spondyloarthritis patients with and without intestinal symptoms-searching for discriminating biomarkers. Cent. Eur. J. Immunol..

[8] Fragoulis GE (2019). Inflammatory bowel diseases and spondyloarthropathies: From pathogenesis to treatment. World J. Gastroenterol..

[9] Mielants H, Veys EM, Cuvelier C, De Vos M (1988). Ileocolonoscopic findings in seronegative spondylarthropathies. Br. J. Rheumatol..

[10] Speca S, Dubuquoy L (2017). Chronic bowel inflammation and inflammatory joint disease: Pathophysiology. Jt. Bone Spine.

[11] Stebbings S (2002). Comparison of the faecal microflora of patients with ankylosing spondylitis and controls using molecular methods of analysis. Rheumatology (Oxford).

[12] Costello ME (2015). Brief Report: Intestinal dysbiosis in ankylosing spondylitis. Arthritis. Rheumatol..

[13] Zhang L (2018). The association of HLA-B27 and *Klebsiella pneumoniae* in ankylosing spondylitis: a systematic review. Microb. Pathog..

[14] Tejera B, Grados D, Martinez-Morillo M, Roure S (2012). Artritis reactiva por *Blastocistys hominis* [Reactive arthritis caused by *Blastocystis hominis*]. Reumatol. Clin..

[15] Alamlih L, Abufaied M, Al-Allaf AW (2020). An unusual cause of reactive arthritis with urticarial: A case report. Qatar Med. J..

[16] Hay EM, Winfield J, McKendrick MW (1987). Reactive arthritis associated with *Cryptosporidium* enteritis. Br. Med. J. (Clin. Res. Ed.).

[17] Shepherd RC, Smail PJ, Sinha GP (1989). Reactive arthritis complicating cryptosporidial infection. Arch. Dis. Child..

[18] McGill PE (1995). Rheumatic syndromes associated with parasites. Baillieres Clin. Rheumatol..

[19] Inman RD (1987). Arthritis and enteritis-an interface of protean manifestations. J. Rheumatol..

[20] Schirmer M (1998). *Entamoeba hartmanni*: A new causative agent in the pathogenesis of reactive arthritis?. Rheumatol. Int..

[21] Than-Saw *et al*. Isolation of *Entamoeba histolytica* from arthritic knee joint. *Trop Geogr Med*. **44**, 355–358 (1992). 1295146

[22] Woo P, Panayi GS (1984). Reactive arthritis due to infestation with *Giardia lamblia*. J. Rheumatol..

[23] Shaw RA, Stevens MB (1987). The reactive arthritis of giardiasis. A case report. JAMA.

[24] González-Gay, M. A., Cereijo, M. J., Agüero, J. J. & Sánchez-Andrade, A. Artritis reactiva a *Giardia lamblia* [Arthritis reactive to *Giardia lamblia*]. *Med. Clin. (Barc.)*. **26**, 359 (1992). 1435014

[25] Bocanegra TS, Espinoza LR, Bridgeford PH, Vasey FB, Germain BF (1981). Reactive arthritis induced by parasitic infestation. Ann. Intern Med..

[26] Richter J (2006). Arthritis associated with *Strongyloides stercoralis* infection in HLA B-27-positive African. Parasitol. Res..

[27] Sert M, Ozbek S, Paydas S, Yaman A (2007). Is there any relationship between *Toxoplasma* infection and reactive arthritis?. J. Postgrad. Med..

[28] Cantey PT (2011). Study of nonoutbreak giardiasis: novel findings and implications for research. Am. J. Med..

[29] Painter JE, Collier SA, Gargano JW (2017). Association between *Giardia* and arthritis or joint pain in a large health insurance cohort: Could it be reactive arthritis?. Epidemiol. Infect..

[30] Johne B (1997). Functional and clinical aspects of the myelomonocyte protein calprotectin. Mol. Pathol..

[31] Simioni J (2019). Fecal Calprotectin, gut Inflammation and spondyloarthritis. Arch. Med. Res..

[32] Bjarnason I (2003). Subclinical intestinal inflammation and sacroiliac changes in relatives of patients with ankylosing spondylitis. Gastroenterology.

[33] Konikoff M, Denson LA (2006). Role of fecal calprotectin as a biomarker of intestinal inflammation in inflammatory bowel disease. Inflamm. Bowel Dis..

[34] Klingberg E, Carlsten H, Hilme E, Hedberg M, Forsblad-d'Elia H (2012). Calprotectin in ankylosing spondylitis-frequently elevated in feces, but normal in serum. Scand. J. Gastroenterol..

[35] Matzkies FG (2012). Markers of intestinal inflammation in patients with ankylosing spondylitis: A pilot study. Arthritis. Res. Ther..

[36] Klingberg E (2017). A longitudinal study of fecal calprotectin and the development of inflammatory bowel disease in ankylosing spondylitis. Arthritis. Res. Ther..

[37] Moreno MJ, Moreno MJ, Linares LF (2019). Relationship between fecal calprotectin, anti-*Saccharomyces cerevisiae* antibodies and other markers of disease activity in patients with spondyloarthritis. Reumatol. Clin. (Engl. Ed.)..

[38] Ma Y (2020). Calprotectin in spondyloarthritis: A systematic review and meta-analysis. Int. Immunopharmacol..

[39] Akoglu H (2018). User's guide to correlation coefficients. Turk. J. Emerg. Med..

[40] Poulsen CS, Stensvold CR (2016). Systematic review on *Endolimax nana*: a less well studied intestinal ameba. Trop. Parasitol..

[41] Burnstein SL, Liakos S (1983). Parasitic rheumatism presenting as rheumatoid arthritis. J. Rheumatol..

[42] Bermúdez A, Flórez O, Bolaños MV, Medina JJ, Salcedo-Cifuentes M (2013). Enteroparasitismo, higiene y saneamiento ambiental en menores de seis comunidades indígenas. Cali-Colombia [Enteroparasitism, hygiene and environmental sanitation in under-aged from six indigenous communities. Cali-Colombia]. Rev. Salud Publica..

[43] Hernández, P. C. *et al.* Intestinal parasitic infections and associated factors in children of three rural schools in Colombia. A cross-sectional study. *PLoS One*. **14**, e0218681; 10.1371/journal.pone.0218681 (2019). 10.1371/journal.pone.0218681 PMC6619675 31291262

[44] Vidal S, Toloza L, Cancino B (2010). Evolución de la prevalencia de enteroparasitosis en la ciudad de Talca, Región del Maule, Chile. Rev. Chilena Infectol..

[45] Barra M, Bustos L, Ossa X (2016). Inequality in the prevalence of intestinal parasitic infections among schoolchildren from urban and rural schools. Rev. Med Chile..

[46] Freites A, Colmenares D, Pérez M, García M, Díaz de Suárez O (2009). Infección por *Cryptosporidium sp* y otros parásitos intestinales en manipuladores de alimentos del estado Zulia, Venezuela. Invest. Clin..

[47] Faria CP (2017). Geospatial distribution of intestinal parasitic infections in Rio de Janeiro (Brazil) and its association with social determinants. PLoS Negl. Trop. Dis..

[48] Ignacio CF (2017). Socioenvironmental conditions and intestinal parasitic infections in Brazilian urban slums: A cross-sectional study. Rev. Inst. Med. Trop. Sao Paulo..

[49] Amin OM (2002). Seasonal prevalence of intestinal parasites in the United States during 2000. Am. J. Trop. Med. Hyg..

[50] Church C, Neill A, Schotthoefer AM (2010). Intestinal infections in humans in the Rocky Mountain region, United States. J. Parasitol..

[51] Belkessa S (2021). Prevalence and Clinical Manifestations of *Giardia intestinalis* and Other Intestinal Parasites in Children and Adults in Algeria. Am. J. Trop. Med. Hyg..

[52] Graczyk TK (2005). The association of *Blastocystis hominis* and *Endolimax nana* with diarrheal stools in Zambian school-age children. Parasitol. Res..

[53] Sarkari B, Hosseini G, Motazedian MH, Fararouei M, Moshfe A (2016). Prevalence and risk factors of intestinal protozoan infections: A population-based study in rural areas of Boyer-Ahmad district, Southwestern Iran. BMC Infect. Dis..

[54] Dudlová A (2018). Prevalence of non-pathogenic types of gastrointestinal protozoa in population in Slovakia and their potential importance in the aspect of public health. Acta Parasitol..

[55] Légeret C, Rüttimann C, Fankhauser H, Köhler H (2021). Parasitic infections in Swiss children: Are we overtesting?. BMC Gastroenterol..

[56] De Melo, G. B., Bosqui, L. R., Da Costa, I. N., De Paula, F. M. & Gryschek, R. Current status of research regarding *Blastocystis sp*., an enigmatic protist, in Brazil. *Clinics*. ***76***, e2489. 10.6061/clinics/2021/e2489 (2021). 10.6061/clinics/2021/e2489 PMC8240786 34231705

[57] Ning, C. Q., Hu, Z. H., Chen. J. H., Ai, L. & Tian, L. G. Epidemiology of *Blastocystis* infection from 1990 to 2019 in China. *Infect Dis Poverty*. **9**, 168. 10.1186/s40249-020-00779-z (2020). 10.1186/s40249-020-00779-z PMC7772921 33380335

[58] Scanlan PD (2014). The microbial eukaryote *Blastocystis* is a prevalent and diverse member of the healthy human gut microbiota. FEMS Microbiol. Ecol..

[59] Lhotská, Z. *et al*. A study on the prevalence and subtype diversity of the intestinal protist *Blastocystis sp*. In a gut-healthy human population in the Czech Republic. *Front. Cell Infect. Microbiol*. **10**, 544335; 10.3389/fcimb.2020.544335 (2020). 10.3389/fcimb.2020.544335 PMC7573152 33123491

[60] Petersen AM (2013). Active ulcerative colitis associated with low prevalence of *Blastocystis* and *Dientamoeba fragilis* infection. Scand. J. Gastroenterol..

[61] Andersen, L. O., Bonde, I., Nielsen, H. B. & Stensvold, C. R. A retrospective metagenomics approach to studying *Blastocystis*. *FEMS Microbiol Ecol*. **91**, 7. 10.1093/femsec/fiv072 (2015). 10.1093/femsec/fiv072 26130823

[62] Tito RY (2019). Population-level analysis of *Blastocystis* subtype prevalence and variation in the human gut microbiota. Gut.

[63] Nieves-Ramírez, M. E. *et al*. Asymptomatic intestinal colonization with protist *Blastocystis* is strongly associated with distinct microbiome ecological patterns. *mSystems*. **3**, e00007-18; 10.1128/mSystems.00007-18 (2018). 10.1128/mSystems.00007-18 PMC6020473 29963639

[64] Ibrahim, H. S., Salem, A. I., Ahmed, N. M. A. E. R. & El-Taweel, H. A. Pre-and post-treatment evaluation of Intestinal Inflammation in *Giardia* and *Blastocystis* infected children: a community-based study. *J Parasit Dis*. 10.21203/rs.3.rs-308755/v1 (2021). 10.1007/s12639-021-01398-7 PMC8556472 34789986

[65] Billy, V. *et al*. *Blastocystis* colonization alters the gut microbiome and, in some cases, promotes faster recovery from induced colitis. *Front Microbiol.* **12**, 641483. 10.3389/fmicb.2021.641483 (2021). 10.3389/fmicb.2021.641483 PMC8058373 33897648

[66] Coskun A (2016). *Blastocystis* in ulcerative colitis patients: genetic diversity and analysis of laboratory findings. Asian Pac. J. Trop. Med..

[67] Javaherizadeh H, Khademvatan S, Soltani S, Torabizadeh M, Yousefi E (2014). Distribution of haematological indices among subjects with *Blastocystis hominis* infection compared to controls. Prz Gastroenterol..

[68] Maiden L, Thjodleifsson B, Theodors A, Gonzalez J, Bjarnason I (2005). A quantitative analysis of NSAID-induced small bowel pathology by capsule enteroscopy. Gastroenterology.

[69] Bjarnason I, Hayllar J, MacPherson AJ, Russell AS (1993). Side effects of nonsteroidal anti-inflammatory drugs on the small and large intestine in humans. Gastroenterology.

[70] Lamot, L. *et al*. The increased levels of fecal calprotectin in children with active enthesitis related arthritis and MRI signs of sacroiliitis: The results of a single center cross-sectional exploratory study in juvenile idiopathic arthritis patients. *Front. Med*. ***8***, 650619. 10.3389/fmed.2021.650619 (2021). 10.3389/fmed.2021.650619 PMC7982855 33763437

[71] Garrett S (1994). A new approach to defining disease status in ankylosing spondylitis: The Bath Ankylosing Spondylitis Disease Activity Index. J Rheumatol..

[72] Lukas, C. *et al*. Assessment of SpondyloArthritis International Society. Development of an ASAS-endorsed disease activity score (ASDAS) in patients with ankylosing spondylitis. *Ann Rheum Dis*. **68**, 18–24 (2009). 10.1136/ard.2008.094870 18625618

[73] Menu, E. *et al*. Evaluation of two DNA extraction methods for the PCR-based detection of eukaryotic enteric pathogens in fecal samples. *BMC Res Notes*. **11**, 206. 10.1186/s13104-018-3300-2 (2018). 10.1186/s13104-018-3300-2 PMC5869780 29587846

[74] Hamzah Z, Petmitr S, Mungthin M, Leelayoova S, Chavalitshewinkoon-Petmitr P (2006). Differential detection of *Entamoeba histolytica, Entamoeba dispar,* and *Entamoeba moshkovskii* by a single-round PCR assay. J. Clin. Microbiol..

[75] Mejia R (2013). A novel, multi-parallel, real-time polymerase chain reaction approach for eight gastrointestinal parasites provides improved diagnostic capabilities to resource-limited at-risk populations. Am. J. Trop. Med. Hyg..

[76] Stensvold, C. R., Ahmed, U. N., Andersen, L. O. & Nielsen HV. Development and evaluation of a genus-specific, probe-based, internal-process-controlled real-time PCR assay for sensitive and specific detection of *Blastocystis* spp. *J Clin Microbiol.* **50**, 1847–1851 (2012). 10.1128/JCM.00007-12 PMC3372105 22422846

