# Supplementary material for: Decreased fecal calprotectin levels in Spondyloarthritis patients colonized by Blastocystis spp
Source: Sci Rep. 2022 Sep 23;12:15840. doi: 10.1038/s41598-022-18308-3 (PMC9508226; doi:10.1038/s41598-022-18308-3)
Supplement: Supplementary file 1 — Supplementary Information. [file 41598_2022_18308_MOESM1_ESM.docx]

**Decreased fecal calprotectin levels in Spondyloarthritis patients colonized by *Blastocystis* spp*.***

Jacqueline Chaparro-Olaya^a^*****, Liliana Morales^a^, Moisés David León Falla^b^, Paula C. Hernández^a^, Wilson Bautista-Molano^c^, Alejandro Ramos-Casallas^b^, Juliette de Ávila^b^, Juan Manuel Bello-Gualtero^d^, Fabián Cortés Muñoz^e^, Consuelo Romero-Sánchez^f^.

### **Affiliations**

**^a^** Laboratorio de Parasitología Molecular, Vicerrectoría de Investigaciones, Universidad El Bosque. Bogotá, Colombia.

**^b^** Cellular and Molecular Immunology Group (InmuBo), Universidad El Bosque. Bogotá, Colombia.

**^c^** Cellular and Molecular Immunology Group (InmuBo), Universidad El Bosque. Clinical Immunology Group, School of Medicine, Hospital Militar Central. Bogotá, Colombia.

**^d^** Rheumatology and Immunology Department & Clinical Immunology Group, School of Medicine, Hospital Militar Central. Bogotá, Colombia.

**^e^** Vicerrectoría de Investigaciones. Universidad El Bosque. Bogotá, Colombia.

**^f^** Cellular and Molecular Immunology Group InmuBo, Universidad El Bosque. Rheumatology and Immunology Department & Clinical Immunology Group, School of Medicine, Hospital Militar Central. Bogotá, Colombia.

### ***Corresponding author**

E-mail address: chaparrojacqueline@unbosque.edu.co. Present address: Laboratorio de Parasitología Molecular. Instituto de Biología Molecular. Universidad de El Bosque. Edificio O. Segundo piso. Avenida Cra. 9 No. 131 A – 02. Bogotá. Colombia. South America. Telephone and fax numbers: Telephone 57(1) 6489000. Ext. 1522.

**Figure S1. Microscopic examination of intestinal parasites.**

(A) *Blastocystis* spp. (B) *Chilomastix mesnili*. (C) *Endolimax nana*. (D) *Entamoeba coli*. (E) *Entamoeba histolytica/dispar/moshkovskii* complex. (F) *Giardia intestinalis*. Direct wet mount. Lugol's Iodine stain (400X).


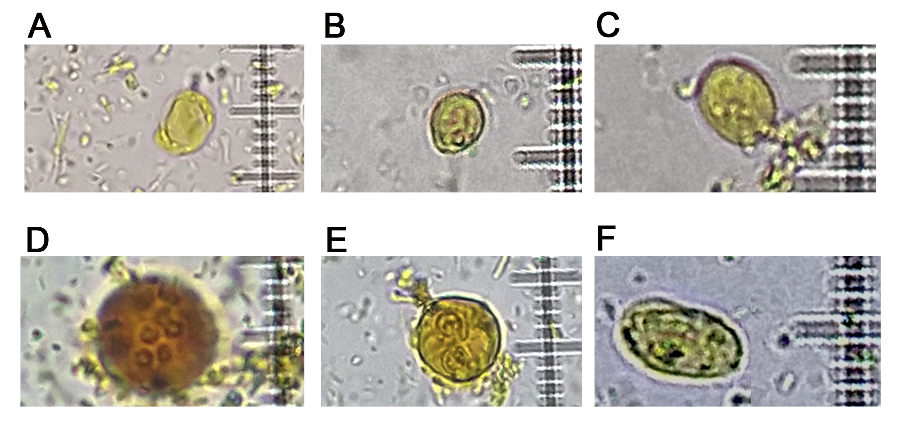


**Table S1. Intestinal parasites found in SpA-patients and controls.**

| **Participants** | | **Group** | ***Endolimax nana*** | ***Blastocystis***  **spp*.*** | ***Entamoeba histolytica*** | ***E. dispar / moshkovskii*** | ***Giardia intestinalis*** | ***Chilomastix mesnili*** | ***Entamoeba***  ***coli*** | ***Iodamoeba bütschlii*** |
| --- | --- | --- | --- | --- | --- | --- | --- | --- | --- | --- |
| **(n)** | **%** |  |  |  |  |  |  |  |  |  |
| **Monoinfection** | | | | | | | | | | |
| 14 | 27.5 | SpA-patients | ⚫ |  |  |  |  |  |  |  |
| 9 | 18.0 | Control | ⚫ |  |  |  |  |  |  |  |
| **Infection with two parasites** | | | | | | | | | | |
| 26 | 51.0 | SpA-patients | ⚫ | ⚫ |  |  |  |  |  |  |
| 28 | 56.0 | Control | ⚫ | ⚫ |  |  |  |  |  |  |
| 1 | 1.96 | SpA-patients | ⚫ |  | ⚫ |  |  |  |  |  |
| 1 | 2.00 | Control | ⚫ |  | ⚫ |  |  |  |  |  |
| 1 | 2.00 | Control | ⚫ |  |  |  |  |  | ⚫ |  |
| 1 | 1.96 | SpA-patients | ⚫ |  |  |  |  |  | ⚫ |  |
| 1 | 1.96 | SpA-patients | ⚫ |  |  |  |  | ⚫ |  |  |
| **Infection with three parasites** | | | | | | | | | | |
| 1 | 2.00 | Control | ⚫ | ⚫ | ⚫ |  |  |  |  |  |
| 1 | 1.96 | SpA-patients | ⚫ | ⚫ | ⚫ |  |  |  |  |  |
| 1 | 1.96 | SpA-patients | ⚫ | ⚫ |  | ⚫ |  |  |  |  |
| 2 | 4.00 | Control | ⚫ | ⚫ |  |  | ⚫ |  |  |  |
| 1 | 2.00 | Control | ⚫ | ⚫ |  |  |  | ⚫ |  |  |
| 5 | 10.0 | Control | ⚫ | ⚫ |  |  |  |  | ⚫ |  |
| 4 | 7.84 | SpA-patients | ⚫ | ⚫ |  |  |  |  | ⚫ |  |
| **Infection with four parasites** | | | | | | | | | | |
| 1 | 2.00 | Control | ⚫ | ⚫ |  | ⚫ |  |  | ⚫ |  |
| 1 | 2.00 | Control | ⚫ | ⚫ |  | ⚫ |  |  |  | ⚫ |
| 1 | 1.96 | SpA-patients | ⚫ | ⚫ | ⚫ |  | ⚫ |  |  |  |
| 1 | 1.96 | SpA-patients | ⚫ | ⚫ | ⚫ |  |  | ⚫ |  |  |

**Table S2. Gastrointestinal symptoms in SpA-patients colonized by *Blastocystis* spp.**

(*) Z test of proportion differences.

|  | **With *Blastocystis* spp.**  **n = 26** | **Without *Blastocystis* spp.**  **n = 14** | ***p* value*** |
| --- | --- | --- | --- |
| **Gastrointestinal symptoms (n - %)** | | | |
| Diarrhea lasting more than 4 weeks | 12 - 46.2 | 8 - 57.1 | 0.5108 |
| Blood in stool | 3 - 11.5 | 4 - 28.6 | 0.1744 |
| Mucus in stool | 6 - 23.1 | 1 - 7.1 | 0.2040 |
| Abdominal pain | 16 - 61.5 | 11 - 78.6 | 0.2708 |
| Abdominal distention | 15 - 57.7 | 11 - 78.6 | 0.1862 |

**Table S3. Primers and probes used to identify *Enterobacteriaceae, G. intestinalis*, *Blastocystis* spp. and *E. histolytica.***

| **Organism** | **Detection**  **method** | **Target gene** | **Primers and probes sequences (5´→ 3´)** | **Ref.** |
| --- | --- | --- | --- | --- |
| *Enterobacteria* | qPCR | 16S rRNA | TTB_Forward: AGAGTTTGATCMTGGCTCAG  TTB_Reverse: TTACCGCGGCKGCTGGCAC  TTB38K: FAM-CCAKACTCCTACGGGAGGCAGCAG-BHQ1 | 73 |
| *G. intestinalis* | qPCR | 18S rRNA | Giard_16S_F: CATGCATGCCCGCTCA  Giard_16S_R: AGCGGTGTCCGGCTAGC  Giard_16S: FAM-AGGACAACGGTTGCAC-BHQ1 | 75 |
| *Blastocystis* spp*.* | qPCR | 18S rRNA | Blasto_18S_F: GGTCCGGTGAACACTTTGGATTT  Blasto_18S_R: CCTACGGAAACCTTGTTACGACTTCA  Blasto_18S: FAM-TCGTGTAAATCTTACCATTTAGAGGA-BHQ1 | 76 |
| *E. histolytica* | Nested  PCR | 18S rRNA | First reaction  E.histo-18S-F: GTTTGTATTAGTACAAAATGGCCAATTC  EhR: GATCTAGAAACAATGCTTCTCT | 75  74 |
|  |  |  | Second reaction  EntaF: ATGCACGAGAGCGAAAGCAT  EhR: GATCTAGAAACAATGCTTCTCT | 74 |
